# Neutrophil Extracellular Traps in Central Nervous System Diseases

**DOI:** 10.2174/011570159X357211250530095758

**Published:** 2025-10-22

**Authors:** Junang Zhu, Hui Zhu, Wanting Hou, Jing-yao Liu

**Affiliations:** 1 Department of Neurology, The First Hospital, Jilin University, Changchun, China;; 2 Medical College of Yanbian University, Yanbian University, Yanji, China

**Keywords:** Neutrophil extracellular traps, stroke, multiple sclerosis, central nervous system, blood-brain barrier, equilibrium

## Abstract

Neutrophil Extracellular Traps (NETs) are complexes containing DNA fibrils and antimicrobial peptides that are released by neutrophils in response to pathogen stimulation. At the time of their discovery, the neutrophil extracellular trap contained active substances such as Neutrophil Elastase (NE) and myeloperoxidase (MPO). Although NETs were initially thought to be a means for the innate immune system to fight microbial invasion, now they have been observed to have a broader impact throughout the body. In recent studies, NETs have been linked to several neurological disorders and have been found to have varying roles in a number of diseases. In addition to their role in thrombosis, NETs have been identified in various autoimmune diseases. NETs play a significant role in the body when they are produced at the correct time and place; however, when the generation and removal of NETs are out of equilibrium, there can be important implications for human health. Here, the impact of NETs is reviewed in various neurological disorders and their potential clinical applications.

## 1 INTRODUCTION

NETs were first described in 2004 when researchers found that human neutrophils exposed to Phorbol Myristate Acetate (PMA) exhibited a form of death that was neither necrotic nor apoptotic; the neutrophils were noted to shed granules and chromatin to form extracellular fibers [1]. NETs are extracellular fibrils that are shed as a result of neutrophil stimulation. In a study of NETs, histones were found to be the major protein components, followed by granzymes and peptides, which include Neutrophil Elastase (NE), myeloperoxidase (MPO), histone G, Leukocyte Proteinase 3 (PR3), lactoferrin, gelatinase, lysozyme C, calprotectin, neutrophil defensin, and calcium protease [2]. This particular pathway of cell death has been found to be responsible for several cellular processes and is accompanied by the generation of neutrophil extracellular traps, a process referred to as NETosis [3]. The mechanisms underlying early NETosis have not been completely elucidated, but the form of neutrophil death induced by PMA has been studied in detail, and many of the mechanisms leading to NET formation have been discovered. PMA can directly couple to protein kinase C, which results in the release of intracellular calcium ions, activation of the cellular RAF-MEK-ERK pathway, and the generation of reactive oxygen species within cells [4]. Subsequently, NE and MPO are mobilized and transported to the nucleus, where they cleave histones of the nucleus, leading to chromatin densification [5]. Furthermore, because of the increased concentration of intracellular calcium ions, the protein arginine deiminase 4 (PAD4) peptide is activated; this facilitates the conversion of arginine from histones to citrulline, which also reduces the density of chromatin within the nucleus [6]. Also, when the cell membrane ruptures, releasing cell contents, and forming NETs [7].

When NETs were initially discovered, they were thought to represent a means for the human innate immune system to fight against microbial invasion. The net structure of NET represents a good barrier to prevent the spread of pathogens in the body; however, if the number and location of the net structures are not strictly controlled or removed in a timely manner, NETs can have serious adverse consequences for the body. On the one hand, the NE, MPO, and reactive oxygen species produced in association with the formation of NETs can damage the body; on the other hand, long-term exposure to histones and other substances associated with NETs can cause an autoimmune reaction [1]. Moreover, NETs play an important role in the development of various diseases, such as systemic lupus erythematosus [8], thrombosis [9], and atherosclerosis [10]. In this review, we summarize the recent developments in our understanding of the role of NETs in several central nervous system (CNS) diseases.

## 2 STROKE AND NETs

Stroke is the leading cause of death, disability, and dementia worldwide, and its incidence increases with aging [11]. NETs play an important role in vascular plaque formation. When human neutrophils are exposed to cholesterol crystals, NETosis is spontaneously triggered, leading to NET production and promoting intravascular plaque formation [12]. Meanwhile, a study by Essig *et al.* of thrombosis observed during malignant tumors in 37 patients with acute ischemic stroke showed that neutrophils and NETs are important components of brain thrombosis and make a vital contribution to the structural complexity of the thrombus [13]. This was confirmed by Genchi, who showed that NETs were widely present in ischemic stroke thrombi and that the NET content correlated significantly with stroke etiology, with significantly higher levels of NETs in cardiac emboli than in large atherosclerotic thrombi [14]. A study by Cha *et al*. from Yonsei University revealed that neutrophil formation and recruitment in arterial thrombi are time-dependent and that the neutrophil fraction in the thrombi of stroke patients appears to be associated with atrial fibrillation and red blood cell fraction [15]. In patients not receiving statins or anti-thrombotic drugs, a positive correlation exists between the vulnerable nature of vascular plaques and the level of plasma NETs [16]. In 2020, Staessens *et al*. described a case report of a patient with a thrombus that had an atypical structure and was very difficult to remove; this supports the hypothesis that NETs are involved in thrombosis, making the removal of the thromboses difficult [17].

Severe stenosis of the carotid arteries is a common cause of strokes. A recent study showed that NET levels are significantly higher in patients with symptomatic carotid stenosis than those in non-symptomatic and healthy subjects; therefore, NET inhibition may be a potential biomarker and treatment target for recurrent stroke in severe carotid stenosis. Furthermore, in patients with symptomatic carotid stenosis, higher levels of neutrophil-platelet aggregates increase the formation of NETs [18]. NETs may also enhance coagulation activity in patients with carotid stenosis by releasing tissue factors that block the vascular endothelial barrier [18]. The study also found that the differential expression of NETs upstream and downstream of carotid plaques was associated with circulating levels of anti-ApoA-1 IgG [19]. In addition, the results of a Japanese study also demonstrated an association of NETs in carotid plaque vulnerability. PAD4 expression in carotid plaques and the neutrophil-to-lymphocyte ratio in peripheral blood were significantly associated with ulcer formation. It was also shown that PAD4 is an indicator of NETs, suggesting a role for NETs in the instability of carotid plaques and that PAD4 could be used as a biomarker for vulnerable plaques [20]. A study from the University of Rotterdam in the Netherlands concluded that carotid plaque vulnerability was positively associated with plasma NET levels [16, 21], and this association occurred only in patients who were not treated with statins or antiplatelet aggregating agents [16]. A number of studies have shown that the inner layer of the carotid plaque is more accessible than the outer layer and that part of the plaque can separate and migrate with the blood to other sites [22]. Furthermore, the inner layer of the carotid plaque plays a key role in plaque formation, with the aggregation of extracellular traps in this region effectively promoting platelet and lipid accumulation and increasing the size of the plaque [23].

The role of NETs in ischemic stroke is not limited to the narrowing of blood vessels or plaque formation. They also play an important role in the damage of brain tissue after cerebral infarction. In ischemic stroke, injury does not result solely from the initial damage to brain tissue caused by a blood clot preventing blood flow to the brain. Injury continues due to the subsequent inflammatory response caused by the infiltration of inflammatory cells into the damaged tissue. Immediately following ischemic stroke, neutrophils infiltrate the damaged brain tissue and promote an inflammatory response within the damaged brain tissue through a variety of mechanisms. Kim *et al.* found that a danger-associated molecular pattern (DAMP) molecule, high-mobility group box-1 (HMGB1) protein, is involved in neuronal inflammatory damage after ischemic stroke. On the one hand, HMGB1 induces the formation of citrullinated histone H3 (CitH3), which in turn causes neutrophils to generate NETs; on the other hand, when neutrophils generate NETs, HMGB1 is released from the cell and further recruits and activates other neutrophils, exacerbating neuronal damage [24]. MMP-9 contained in NETs can disrupt the blood-brain barrier by degrading matrix proteins, thus allowing more neutrophils to enter the CNS [25]. And NO mediates the production of MMP-9 intracellularly, so Calabrese *et al.* suggested that regulating NO in the CNS could be a potential therapeutic target for neuroprotection [26]. In addition, in an experimental study, Kim *et al.* found that ATP enhances the induction of CitH3 by PAD4, which in turn promotes the formation of NETs; they also found that ATP, similar to HMGB1, mediates a vicious cycle between NETosis and neuronal damage, aggravating inflammation and brain damage [27].

Other studies have shown that thrombi CitH3 levels are significantly higher in patients with hyperglycemia than those with normoglycemia. Studies using a model of middle cerebral artery infarction in hyperglycemic mice found that the hyperglycemic model was associated with higher levels of NETs and a larger cerebral infarct volume [28]. Neovascularization and vascular remodeling play key roles in brain recovery after stroke. Increased PAD4 expression, increased NET production, concomitant decreased neovascularization, and blood-brain barrier damage have been identified in ischemic brain tissue; thus, the production of NETs impairs vascular remodeling during stroke recovery [29]. Calabrese *et al.* proposed that the Keap1/Nrf2/ARE pathway has a neuroprotective effect that also reduces neuronal cell damage by NETs [30]. *In vitro* studies in mice have shown that the deletion of the Nrf2 gene leads to the expansion of infarct size and damage to neural tissue 7 days after ischemia [31]. The production of NETs by neutrophils also generates large amounts of inflammatory factors such as peroxides, which activate the Keap1/Nrf2/ARE pathway to produce reduced glutathione and thereby protect neurons.

t-PA (tissue-type fibrinogen activator) is currently the only approved thrombolytic therapy for stroke, with fibrin being a major component of ischemic stroke thrombosis. Ducroux *et al.* showed that NETs create a mesh scaffold structure within a thrombus, making it more resistant to mechanical and enzymatic damage and that the combination of t-PA and DNase 1 accelerates thrombolysis compared with t-PA or DNase 1 alone [32]. In a mouse model of cerebral ischemia-reperfusion, the combined use of DNase 1 and t-PA significantly reduced infarct volume compared to t-PA alone [32]. These results suggest that high NET levels within a thrombus may be the cause of resistance to reperfusion, but the strategy of combining DNase 1 with t-PA needs further study. It has recently been shown that NETs do not play a major role in the formation of fibrin-rich thrombi, indicating that DNase 1 lacks a lytic effect on fibrin-rich thrombi; this strongly suggests that the pharmacological modulation of NETs in the acute phase of stroke may be a promising strategy for brain injury repair in ischemic conditions independent of thrombus type [33].

The role of NETs in stroke is currently being clarified. Cai *et al.* have proposed a new therapeutic approach following experimental results revealing that N2-type neutrophils assist the clearance of neutrophils by macrophages with less damage to ischemic neurons. Based on this conclusion, Wei *et al.* suggested that directing neutrophils toward the N2 phenotype may be a promising therapeutic approach for ischemic stroke [34]. Huang *et al.* demonstrated that edaravone dextroorbate (EDA.B) significantly improves neurological function and cerebral blood flow after experimental stroke by reducing serum levels of NETs. Moreover, EDA.B exerts neuroprotective effects and improves blood-brain barrier permeability after acute ischemic stroke [35]. Another recent study showed that neonatal NET Inhibitory Factor (nNIF) improves long-term neurological and motor functions in mice with stroke, demonstrating the therapeutic potential of nNIF for stroke [36]. The finding by Di Rosa *et al.* that hydroxytyrosol in olive oil activates the Nrf2-antioxidant response element pathway, thereby protecting biomolecules from oxidative damage, could provide an idea for mitigating brain tissue damage after stroke [37].

## 3 SUBARACHNOID HEMORRHAGE AND NETs

Neuroinflammation plays an important role in early brain injury following subarachnoid hemorrhage. Microglia are the basic effector cells of the inflammatory response in the CNS and are relevant to the prognosis of subarachnoid hemorrhage [38, 39]. Hanhai *et al.* found that peripheral blood neutrophil levels were much higher in patients with SAH than in healthy individuals and that NETs significantly increased the pro-inflammatory subtype shift of microglia; the results of these experiments indicate that neutrophils release NETs after subarachnoid hemorrhage, promoting the shift of microglia to a pro-inflammatory phenotype, leading to neuroinflammation and worsened outcomes [40]. Neurogenic Pulmonary Edema (NPE) is a serious non-neurological complication following subarachnoid hemorrhage. Previous studies have shown an association between NPE, reduced survival, and poor prognosis [41, 42]. A study by Zeng confirmed that NETs are associated with NPE and that the inhibition of lung NET formation in mice could reduce alveolar interstitial swelling [43]. The same study found that NETs promote a neuroinflammatory response following subarachnoid hemorrhage and that pharmacological inhibition of PAD4-NETs mitigates this inflammatory damage, demonstrating that NETs may be a potential therapeutic target for subarachnoid hemorrhage [43]. In a prospective study, Witsch identified the MPO-DNA complex, a biomarker of NETs, in the peripheral blood of patients with aneurysmal subarachnoid hemorrhage and reported that significantly reduced MPO-DNA complex levels may be an early marker of delayed cerebral ischemia after aneurysmal subarachnoid hemorrhage [44].

## 4 MULTIPLE SCLEROSIS AND NETs

Multiple Sclerosis (MS) is a chronic inflammatory demyelinating disease of the CNS that primarily manifests as a secondary neurodegenerative disease. The pathology of MS is characterized by local microglial and astrocyte activation, infiltration of activated macrophages and lymphocytes, and myelin degradation, all of which cause axonal and neuronal damage. Neutrophils play an important role in MS development. Neutrophils have been shown to possess a unique function in MS, exhibiting increased expression of the activating phenotype toll-like receptor 2 (TLR2) and N-formyl-methionyl-leucyl-phenylalanine (fMLP) receptors [45]. Naegele *et al.* suggest that the chronic inflammatory environment of the body in MS is the basis for inappropriate neutrophil initiation, which leads to enhanced neutrophil activation. They also found that neutrophils were numerous and exhibited an initiating state in patients with relapsing-remitting multiple sclerosis. Significantly higher levels of serum NETs were found, suggesting that NETs may be involved in the pathogenesis of MS [46]. Paryzhak *et al.* have also suggested that NETs degrade circulating immune complexes in MS, thereby exposing the internal sugar epitopes [47].

There is a significant difference in the incidence of MS between males and females, with females being two-three times more likely to be diagnosed with multiple sclerosis than males [48]. A study by Tillack *et al.* found evidence indicating that NETs underlie sex differences in the pathogenesis of MS, with levels of NETs significantly higher in the sera of male patients; furthermore, male patients had a worse prognosis [49]. A transcriptomics study also confirmed sex differences in the pathogenesis of MS [50].

## 5 ALZHEIMER'S DISEASE AND NETs

Alzheimer's disease (AD) is a devastating disease affecting the nervous system [51]. The presence of neuroinflammation in AD is a well-recognized phenomenon, but the underlying mechanism has not been fully elucidated [52]. The role of cells in the circulating immune system of patients with AD has not been studied in detail. However, a study performed at the University of Verona in Italy found neutrophils in the cerebral vasculature and brain parenchyma of patients with AD and experimentally confirmed the ability of neutrophils to invade the brain; the researchers also found NETs in the brain parenchyma [53]. Data from the same study indicated that the suppression of neutrophil function in an AD mice model reduced the neuropathological features of AD and improved memory function in mice that had developed cognitive dysfunction, all of which suggests an important role for neutrophils in the pathogenesis of AD [53, 54].

The complement system has long been recognized as a key factor in innate immunity; it is involved in many inflammatory responses and plays an important role in the regulation and protection of the nervous system. However, the possible role of the complement system in NET generation in AD appears to have been relatively neglected in the literature. Recently, Kretzschmar *et al.* suggested that neutrophils can be attracted to β-amyloid and release NETs by various pro-inflammatory factors, such as the complement allergenic toxin C5a [55]. Their results also showed that plasma and serum concentrations of NETs were higher in patients with AD than in controls [55]. A recent study revealed the cellular origin and localization of a large number of peroxisomal enzymes in the brains of patients with AD. Smyth *et al*. confirmed that neutrophil aggregation in the brains of patients with AD is associated with blood vessels and that neutrophils and NETs are the main source of MPO [56]. NETs in the brain can break down the extracellular matrix, leading to neurotoxicity, which may be caused by the associated proteases contained in NETs such as matrix metallopeptidase 9 (MMP-9) and MPO, and also activate the mitochondrial apoptotic pathway and amplify the inflammatory pathway [57-60].

## 6 BLOOD-BRAIN BARRIER AND NETs

**Fig. (1):**
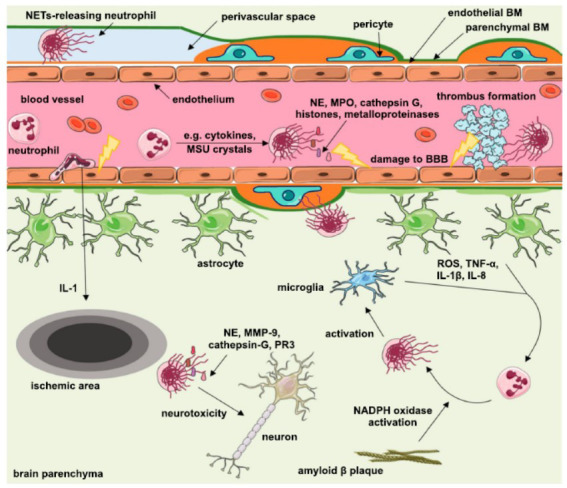
Neutrophil extracellular traps play an important role in intravascular thrombosis and blood-brain barrier disruption. Cytokines, beta-amyloid plaques, reactive oxygen species (ROS), and monosodium urate (MSU) crystals can promote the release of NETs from neutrophils, and NETs in blood vessels can promote thrombosis and carry cytotoxic proteins that directly disrupt the blood-brain barrier. At the same time, extravasated neutrophils can release NETs in the brain parenchyma, and NETs can activate microglia to enhance the release of NETs further.

The blood-brain barrier is a special structure formed by the unique interaction between endothelial cells, pericytes, and astrocyte peduncles of the brain. It is a physical and chemical barrier of the CNS, which acts as a functional barrier and a mechanical barrier preventing most immune cells and immune complexes from entering the CNS [61, 62]. However, most damage to the blood-brain barrier is now considered to be due to two factors: blood-brain barrier rupture and neuroinflammation caused by neurotoxic antibodies [63, 64]. Normally, it is difficult for neutrophils to cross the blood-brain barrier into the CNS; however, neutrophil infiltration of CNS tissues is a well-known phenomenon that occurs in various CNS disease pathologies. In patients with lupus, intercellular adhesion molecule 1 and selectin-E expression are upregulated in astrocytes and microglia associated with the blood-brain barrier, whereas neutrophil cell membranes coincide with the presence of ligands for these adhesion molecules [65]. MMP-9 secreted by neutrophils has been found to be associated with basal collagen degradation and can promote blood-brain barrier breakdown [66]. It has also been shown that NETs contain related proteases such as MMP-9 [59], and thus, it is likely that neutrophils damage the blood-brain barrier by producing NETs. A study by Elmar Pieterse found that excessive NET production prevents the ability of endothelial cells to degrade NETs, and the excess NETs promote vascular leakage and endothelial-to-mesenchymal transition through the degradation of VE-cadherin and the subsequent activation of β-catenin signaling, leading to endothelial cell injury [67]. All these mechanisms can damage the blood-brain barrier, eventually leading to neutrophil entry, which in turn further exacerbates the damage to the blood-brain barrier. After blood-brain barrier damage, neurotoxic mediators enter and damage the CNS. All these mechanisms may play an important role in the development of damage to the blood-brain barrier (Fig. **1**).

## CONCLUSION

In recent years, the role of NETs in CNS diseases has attracted the attention of many researchers. However, current research on NETs in CNS diseases is unbalanced; the role of NETs in stroke has been studied extensively, resulting in a large body of supporting data, but studies of their role in MS, AD, and other diseases are limited, and further research is needed to address this. Recent studies of NETs in spinal cord injury, cerebral arteriovenous malformations, and meningitis have demonstrated increasing interest in the role of NETs in CNS diseases [68-70] as well as corroborating the widespread role of NETs in CNS diseases. Prospects for the clinical application of NETs have also been explored. Several drugs have been found to reduce the production of NETs or promote NET degradation through different mechanisms. The use of such drugs in CNS diseases may have beneficial effects on the prevention or treatment of these diseases. For example, the flavonoid luteolin and carnosic acid inhibit neutrophil extracellular trap production by inhibiting the Raf1-MEK-1-Erk pathway and reactive oxygen species production [71, 72]. Salvia miltiorrhiza significantly inhibits early neutrophil extracellular trap formation by inhibiting myeloperoxidase and NADPH oxidase activities [73]. It has also been found that Xuebijing injection has a significant inhibitory effect on the production of NETs by neutrophils [74, 75]. Moreover, NETs are present in the serum and can be used as circulating markers to predict the occurrence of disease or assess disease severity. We hope that this review will provide new ideas or useful information for subsequent research on NETs in CNS diseases and contribute to progress in the diagnosis and treatment of related CNS diseases.

## AUTHORS’ CONTRIBUTIONS

The authors confirm their contribution to the paper as follows: JYL provided the idea, HZ and WTH reviewed the documents, and JAZ drafted the manuscript. All authors reviewed the results and approved the final version of the manuscript.

## LIST OF ABBREVIATIONS

AD: Alzheimer's Disease
CitH3: Citrullinated Histone H3
CNS: Central Nervous System
DAMP: Danger-associated Molecular Pattern
HMGB1: High-mobility Group Box-1
MMP-9: Matrix Metallopeptidase 9
MPO: Myeloperoxidase
MS: Multiple Sclerosis
NE: Neutrophil Elastase
NETs: Neutrophil Extracellular Traps
NPE: Neurogenic Pulmonary Edema
PAD4: Protein Arginine Deiminase 4
PMA: Phorbol Myristate Acetate
